# Lipid Accumulation in HepG2 Cells Is Attenuated by Strawberry Extract through AMPK Activation

**DOI:** 10.3390/nu9060621

**Published:** 2017-06-16

**Authors:** Tamara Y. Forbes-Hernández, Francesca Giampieri, Massimiliano Gasparrini, Sadia Afrin, Luca Mazzoni, Mario D. Cordero, Bruno Mezzetti, José L. Quiles, Maurizio Battino

**Affiliations:** 1 Dipartimento di Scienze Cliniche Specialistiche ed Odontostomatologiche (DISCO)-Sez. Biochimica, Facoltà di Medicina, Università Politecnica delle Marche, 60131 Ancona, Italy; tamara.forbe@gmail.com (T.Y.F.-H.); f.giampieri@univpm.it (F.G.); m.gasparrini@univpm.it (M.G.); dolla.bihs@gmail.com (S.A.); 2 Área de Nutrición y Salud, Universidad Internacional Iberoamericana (UNINI), Campeche 24040, Mexico; 3 Dipartimento di Scienze Agrarie, Alimentari e Ambientali, Università Politecnica delle Marche, 60131 Ancona, Italy; l.mazzoni@univpm.it (L.M.); b.mezzetti@univpm.it (B.M.); 4 Research Laboratory, Dental School, University of Sevilla, 41009 Sevilla, Spain; mdcormor@us.es; 5 Department of Physiology, Institute of Nutrition and Food Technology “José Mataix”, Biomedical Research Centre, University of Granada, 18000 Granada, Spain; jlquiles@ugr.es; 6 Centre for Nutrition & Health, Universidad Europea del Atlantico (UEA), 39011 Santander, Spain

**Keywords:** strawberry, cholesterol synthesis, fatty acids synthesis, hypolipidemic agent

## Abstract

Regulation of lipid metabolism is essential for treatment and prevention of several chronic diseases such as obesity, diabetes, and cardiovascular diseases, which are responsible for most deaths worldwide. It has been demonstrated that the AMP-activated protein kinase (AMPK) has a direct impact on lipid metabolism by modulating several downstream-signaling components. The main objective of the present work was to evaluate the in vitro effect of a methanolic strawberry extract on AMPK and its possible repercussion on lipid metabolism in human hepatocellular carcinoma cells (HepG2). For such purpose, the lipid profile and the expression of proteins metabolically related to AMPK were determined on cells lysates. The results demonstrated that strawberry methanolic extract decreased total cholesterol, low-density lipoprotein (LDL)-cholesterol, and triglycerides levels (up to 0.50-, 0.30-, and 0.40-fold, respectively) while it stimulated the p-AMPK/AMPK expression (up to 3.06-fold), compared to the control. AMPK stimulation led to the phosphorylation and consequent inactivation of acetyl coenzyme A carboxylase (ACC) and inhibition of 3-hydroxy-3-methylglutaryl-CoA reductase (HMGCR), the major regulators of fatty acids and cholesterol synthesis, respectively. Strawberry treatment also entailed a 4.34-, 2.37-, and 2.47-fold overexpression of LDL receptor, sirtuin 1 (Sirt1), and the peroxisome proliferator activated receptor gamma coactivator 1-alpha (PGC-1α), respectively, compared to control. The observed results were counteracted by treatment with compound C, an AMPK pharmacological inhibitor, confirming that multiple effects of strawberries on lipid metabolism are mediated by the activation of this protein.

## 1. Introduction

Dyslipidemia—a major disorder of lipoprotein metabolism—is one of the main risk factors for cardiovascular diseases (CVDs) including coronary heart disease, heart attacks, strokes, and peripheral vascular disease [1,2,3], which are responsible for most deaths worldwide [4,5]. Elevated total cholesterol, low-density lipoprotein (LDL)-cholesterol, and triglycerides levels are also correlated with type 2 diabetes, insulin resistance [1], and chronic renal disease [6]. Hence, understanding lipid metabolism is essential for the treatment and prevention of all these pathologies.

Recently, the AMP-activated protein kinase (AMPK) has been proposed as a potential therapeutic target for the treatment of several chronic diseases including obesity, type 2 diabetes, and CVDs [7,8]. AMPK is a serine/threonine protein kinase that plays a central role in regulating cellular metabolism and energy balance in mammalian cells [9]. When cellular energy stores are depleted by stimuli such as muscle contraction, hypoxia, or myocardial ischaemia, AMPK is activated. Once activated, AMPK induces ATP generation pathways such as glycolysis, fatty acid oxidation, and lipolysis. On the contrary, anabolic pathways like gluconeogenesis, and cholesterol and protein synthesis are inhibited. The overall effects of this protein—when activated—on the lipid metabolism are to stimulate fatty acid oxidation and to block cholesterol and triglycerides synthesis [9,10].

Of current interest are the effects of naturally occurring compounds in the treatment and/or prevention of particular diseases by activating AMPK. Many of these natural compounds, including epigallocatechin gallate, resveratrol, quercetin, theaflavin, curcumin, berberine, ginsenoside, and caffeic acid phenethyl ester, belong to the polyphenols family [10]. Therefore, it is suggested that polyphenolic compounds from natural sources may be used to activate AMPK.

In this context, strawberries (*Fragaria* × *ananassa*) are one of the richest dietary sources of phytochemicals, mainly represented by flavonoids, hydrolysable tannins, and phenolic acids [11,12].

Scientific evidence indicates the role of this berry in the diminution of oxidative damage and inflammation, which play a critical role in the development of chronic diseases [13]. In different models of obesity/diabetes, it has been demonstrated that whole strawberries or purified anthocyanin supplementation normalize blood glucose levels and limit glucose transport and uptake [14]. In addition, data from in vitro experiments suggest that the bioactive compounds from strawberries can modify the cell membrane composition, functionality, and fluidity, protecting lipid bilayers against oxidative damage [15,16].

In obese and lean C57BL/6 mice models, strawberry supplementation decreases plasma c-reactive protein (CRP) and overall blood glucose concentrations independently of the dietary fat content, demonstrating a protective effect against cardiovascular risk [17]. In addition, some clinical studies observed that strawberry intake ameliorates some atherosclerotic risk factors by reducing circulating levels of vascular cell adhesion molecule 1 [13], and CRP [18,19], decreasing lipid peroxidation [19], and improving lipid profile [13,19,20].

The reduction of lipid levels in plasma can be explained through the ability of strawberry polyphenols to activate the molecular signaling pathways involved in lipid metabolism, but this hypothesis must still be confirmed. The main objective of the present work was to evaluate the in vitro effect of strawberry (cv. Romina) methanolic extract on AMP-activated protein kinase (AMPK) cascade and its possible repercussion on lipid metabolism in human hepatocellular carcinoma cells (HepG2).

## 2. Materials and Methods

### 2.1. Preparation of Strawberry Extract

Strawberry fruits, *Fragaria* × *ananassa* (cv. Romina) were collected in the experimental fields of Azienda Agraria Didattico Sperimentale (Università Politecnica delle Marche, Ancona, Italy). Within 2 h after harvest, whole fruits were stored at −80 °C until time of analyses. For the strawberry extract preparation, 10 g of fruits were added to 100 mL of the extraction solution, consisting of methanol/MilliQ water/concentrated formic acid (80:20:0.1 *v*/*v*), and homogenized using an Ultraturrax T25 homogenizer (Janke & Kunkel, IKA Labortechnik). Extraction was maximized by stirring the suspension for 2 h in the dark at room temperature. The mixture was then centrifuged at 2400× *g* for 15 min in two sequential times, to sediment solids. Supernatants were filtered through a 0.45 µm Minisart filter (PBI International), transferred to 5.0 mL amber glass vials, and stored at −80 °C until analysis. For cellular treatment, the methanolic extract was further concentrated and dried through a rotary evaporator and stored in aliquots at −80 °C.

### 2.2. Strawberry Extract Characterization

#### 2.2.1. Total Phenolic Compounds (TPC) and Flavonoids Content Determination

TPC of the strawberry extract was determined using the Folin-Ciocalteu method, as modified by Slinkard and Singleton [21], while the total flavonoid content was determined through a colorimetric method previously described by Jia et al. [22] and Dewanto et al. [23].

#### 2.2.2. Vitamin C Content Determination

For vitamin C quantification, the extracting solution consisted of MilliQ water containing 5% meta-phosphoric acid and 1 mM ethylenediaminetetraacetic acid. Vitamin C was extracted by sonication of 1 g of frozen strawberries in 4 mL of extracting solution, for the duration of 5 min, after a previous homogenization using an Ultraturrax T25 homogenizer (Janke & Kunkel, IKA Labortechnik, Staufen im Breisgau, Germany) at medium-high speed for 2 min. After the ultrasound assisted extraction, solids were precipitated by centrifugation at 1720× *g* for 10 min at 4 °C, and the supernatant was filtered through a 0.45 µm filter into 1.8 mL high-performance liquid chromatography (HPLC) vials and immediately analyzed as described by Helsper et al. [24] in an HPLC system (Jasco, PU-2089 plus).

#### 2.2.3. Identification and Quantification of Strawberries Anthocyanins

Analysis of anthocyanins was carried out following the method described by Terefe et al. [25]. Anthocyanins were separated from the methanolic extract on an Aqua Luna C18 (2) (250 × 4.6 mm) reverse phase column with a particle size of 5 µm (Phenomenex) protected by a Phenomenex 4.0 × 3.0 mm C18 ODS guard column. The samples were diluted appropriately and filtered using a 0.45 µm filter prior to injection into the HPLC system (Jasco, PU-2089 plus). Anthocyanins were quantified using external Cyanidin-3-*O*-glucoside, Pelargonidin-3-*O*-glucoside, and Pelargonidin-3-*O*-rutinoside calibration curves.

#### 2.2.4. Total Antioxidant Capacity (TAC) Determination

For TAC determination, three different methods were employed: the Ferric Reducing Antioxidant Power (FRAP) method described by Deighton et al. [26]; the 2,2-diphenyl-1-picrylhydrazyl (DPPH) free radical method described by Kumaran and Karunakaran [27]; and the Trolox Equivalent Antioxidant Capacity (TEAC) method described by Re et al. [28].

### 2.3. Cells Culture and Cells’ Lysates Preparation

HepG2 cells were kindly provided by the Biological Research Laboratory of Seville University (Spain), and were grown in Dulbecco’s modified Eagle’s medium (DMEM), supplemented with 10% fetal bovine serum (FBS), 100 IU/mL penicillin, and 100 µg/mL streptomycin at 37 °C with 5% CO_2_.

After treatments with the strawberry dried methanolic extract, compound C, or lovastatin for the indicated times (as detailed below), cells were lysed in a buffer containing 20 mM Tris–HCl (pH 7.5), 0.9% NaCl, 0.2% Triton X-100, and 1% of the protease inhibitor cocktail (Sigma-Aldrich, Milan, Italy) and then stored at −80 °C for further western blot analysis or were lysed in radioimmunoprecipitation assay (RIPA) buffer (Sigma-Aldrich, Milan, Italy) for lipid profile determination. All the analyses were conducted on cells between the third and the sixth passage.

### 2.4. Cytotoxicity Assay

Cells were seeded at a density of 5 × 10^3^ cells/well into 96-well plates and treated with different concentrations of the dried methanolic extract (from 0 to 1 mg/mL) for 24, 48, and 72 h. Therefore, 30 µL of RPMI medium containing 2 mg/mL of 3-(4,5-dimethylthiazol-2-yl)-2,5-diphenyltetrazolium bromide (MTT) were added and cells were incubated for other 2 h. MTT solution was then discarded and 100 µL of dimethyl sulfoxide were added to dissolve the formazan crystals. The level of colored formazan derivative was analyzed on a microplate reader (Thermo Scientific Multiskan EX, Monza, Italy) at a wavelength of 590 nm [29,30]. The viable cells were directly proportional to the formazan production.

### 2.5. Western Blotting Analysis

Equal amounts of protein (75 µg) of each sample were loaded and separated in a 10% acrylamide sodium dodecyl sulfate polyacrylamide gel electrophoresis (SDS/PAGE) and transferred to nitrocellulose membranes (Bio-Rad) by using a Trans-Blot Turbo Transfer System (Bio-Rad Laboratories, Inc., Hercules, CA, USA). Membranes were washed three times with PBS, blocked with 5% non-fat dry blocker (Bio-Rad), and incubated with the primary antibody at 4 °C overnight. All the polyclonal antibodies for the protein of interest were purchased from Santa Cruz Biotechnology, Inc., Dallas, TX, USA. Next, membranes were washed with 5% Tween 20 in Tris HCl buffered saline and incubated with the secondary anti-rabbit immunoglobulin G-Peroxidase antibody (Sigma-Aldrich, Milan, Italy). Protein bands were visualized using Immobilon Western Chemiluminescent Substrate (Millipore Corporation, Billerica, MA, USA), and the protein signals were detected by a Lycor C-Digit Blot Scanner. Quantification of protein expression was made using the software (Image Studio 3.1) provided by the manufacturer of the Blot Scanner (LI-COR Biotechnology, Bad Homburg, Germany).

### 2.6. Mitochondrial Functionality

Oxygen consumption rate (OCR) was measured in real-time using an XF24 Extracellular Flux Analyzer (Seahorse Bioscience, Billerica, MA, USA) according to the manufacturer’s protocol. Cells were seeded at a density of 3 × 10^4^ cells/well in 24-well plates and treated with the strawberry dried methanolic extract for 24 h. Before the running started, the medium was replaced with DMEM (containing 25 mM glucose, 2 mM glutamine, 1 mM sodium pyruvate, and without serum, pH 7.4) and plates were pre-incubated in the absence of CO2 in a XF Prep Station (Seahorse Bioscience, Billerica, MA, USA) at 37 °C. After finishing calibration, the plate containing cells was placed into the XF24 Extracellular Flux Analyzer (Seahorse Bioscience, Billerica, MA, USA) and mitochondrial functionality was evaluated by sequential injection of four compounds that affect bioenergetics, as follows: 55 µL of oligomycin into port A (final concentration 2.5 µg/mL); 61 µL of carbonyl cyanide-4-(trifluoromethoxy) phenylhydrazone (FCCP) into port B (final concentration 2.5 µM); and 68 µL of rotenone/antimycin A into port C (final concentration 1 µM/10 µM). The best concentration of each inhibitor and uncoupler, as well as the optimal cells seeding density were determined in preliminary analyses. A minimum of five wells per treatment were utilized in any given experiment.

### 2.7. Determination of Total Cholesterol, LDL-Cholesterol, and Triglycerides Levels

Total cholesterol, LDL-cholesterol, and triglycerides levels were determined by enzymatic colorimetric kits (Spinreact, St. Esteve d’en Bas, Girona, Spain) with the employment of a microplate reader (Thermo Scientific Microplate Reader, Multiskan^®^, Monza, Italy) coupled to an Ascent software program (Thermo LabSystems Oy, Version 2.6, Navi Mumbai, India).

### 2.8. Determination of Total Lipid Accumulation by Oil Red O Staining

Total lipid accumulation was evaluated according to the method previously described by Liu et al. [31]. Cells were seeded at a density of 1.5 × 10^5^ cells/well in 6-well plates and treated for 24 h with the strawberry dried methanolic extract, compound C, or lovastatin. Subsequently, cells were rinsed twice with PBS and fixed in 4% paraformaldehyde in PBS for 30 min. Then, cells were stained with Oil Red O working solution for 1 h at room temperature and subsequently rinsed with water. The Oil Red O stock solution was prepared by dissolving 0.35 g of Oil Red O (Sigma Aldrich, Milan, Italy) in 100 mL of isopropanol by gentle heating and then cooled and filtered through a 0.45 µm filter. The working solution was prepared by diluting three parts of the stock solution in two parts of MilliQ water (stock solution:MilliQ water; 3:2 *v*/*v*). The cell images were captured with a Leitz Fluovert FU (Leica Microsystems, Wetzlar, Germany) microscope. Lipids appeared red. For quantitative analysis of cellular lipids, 1 mL isopropanol was added to each well of the stained culture plate. The extracted dye was immediately removed by gentle pipetting and its absorbance was measured at 510 nm.

### 2.9. Statistical Analysis

Statistical analyses were performed using STATISTICA software (Statsoft Inc., Tulsa, OK, USA). Data were subjected to one-way analysis of variance for mean comparison, and significant differences among different treatments were calculated according to Tukey’s HSD (honest significant difference) multiple range test. Data are reported as mean ± standard deviation (SD). Differences at *p* < 0.05 were considered statistically significant. All the analyses were performed in triplicate.

## 3. Results

### 3.1. Characterization of Romina Strawberry Extract

The phytochemical composition of the strawberry extract demonstrated that the Romina strawberry cultivar is a significant source of polyphenols (2.64 ± 2.63 mg gallic acid equivalent (GAeq)/g fresh weight (FW)), in particular of flavonoids (1.02 ± 0.87 mg catechin equivalent (CATeq)/g FW) (Table 1). TPC was similar to the values reported by Capocasa et al. [32] and Tulipani et al. [16] for other commercial varieties like Sveva (2.7 mg GAeq/g FW) and Camarosa (2.6 mg GAeq/g FW) and even higher than the values reported for Adria and Alba (1.8 and 2 mg GAeq/g FW, respectively).

Also, the total flavonoids content (TFC) was higher than the values reported for Adria (0.4 mg CATeq/g FW) and Sveva (0.6 mg CATeq/g FW) [16] while the vitamin C content (0.39 ± 0.23 mg/g FW) was within the values reported by Tulipani et al. [33], who indicated that the average vitamin C content in five selected cultivars (Alba, Irma, Patty, Adria, and Sveva) and three advanced selections (AN94.414.52, AN03.338.51, AN00.239.55) of strawberries ranged from 0.23 to 0.47 mg/g FW.

Regarding the anthocyanins reported in Table 1, they were identified through their spectral and chromatographic characteristics and were in correspondence with the main anthocyanins reported for strawberries. The individual quantified value of Pelargonidin-3-*O*-glucoside (the most representative compound of this group) was significantly higher (0.70 ± 0.25 mg/g FW) than the values reported for Sveva, Alba, and Adria [16,33], although lower than the value reported for this cultivar by Diamanti et al. [34]. These results confirmed that the quality of this cultivar is quite good.

Once the strawberry methanolic extract was concentrated and dried, its TPC, TFC, and Pelargonidin-3-*O*-glucoside concentration were 23.44 ± 0.22 mg GAeq/g dried weight (DW), 5.21 ± 0.29 mg CATeq/g DW, and 16.32 ± 5.71 mg/g DW, respectively. In general, the presence of phenolic compounds and vitamin C is often correlated to the antioxidant power of fruits, since these compounds are efficient oxygen radical scavengers. TAC of Romina fruits was determined by three different methods (Table 1). Results confirmed that the Romina strawberry variety not only presents a good nutritional quality but also a high antioxidant capacity.

### 3.2. Effects of Strawberry Extract on HepG2 Cell Viability

**Figure 1:**
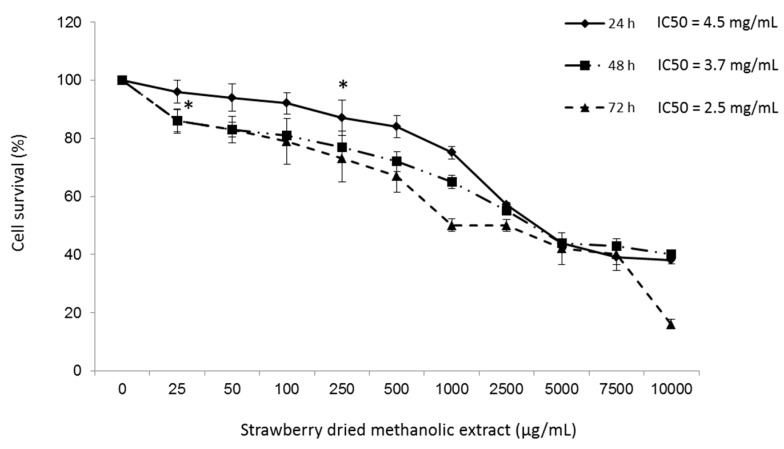
Viability of human hepatocellular carcinoma cells (HepG2) cells after treatment with strawberry dried methanolic extract. Cells were incubated with the indicated concentrations for 24, 48, and 72 h. IC50 indicates the concentration of strawberry dried methanolic extract which reduces the cells viability about 50%. Values are expressed as mean ± SD of three independent experiments (*n* = 3). Asterisk marks indicate the concentrations from which significant differences (*p* < 0.05) were observed compared to the control.

Viability of HepG2 cells treated with different concentrations of strawberry dried methanolic extract was first determined using the MTT assay. The results showed that after 24 h of treatment the strawberry dried methanolic extract at concentrations up to 100 µg/mL did not significantly (*p* < 0.05) cause cell death with respect to control (Figure 1).

Significant cytotoxicity (*p* < 0.05) was observed at higher concentrations or longer treatment times, although in all cases the IC50 was higher than 2.5 mg/mL. Hence, the concentrations 10, 50, and 100 µg/mL of dried methanolic extract were used in subsequent experiments.

### 3.3. Effects of Strawberry Extract on AMPK Activation

**Figure 2:**
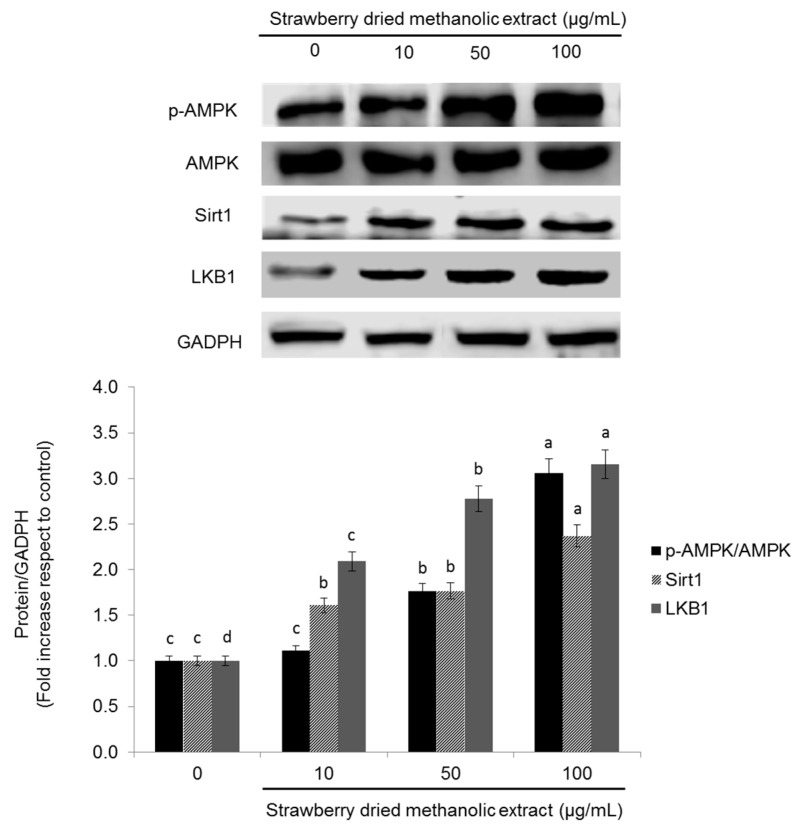
Effects of strawberry dried methanolic extract on p-AMPK, AMPK, Sirt1, and LKB1 expression. HepG2 cells were treated with the indicated concentrations for 24 h. The protein signals were detected by a Lycor C-Digit Blot Scanner and quantification was made using the software Image Studio 3. Values are expressed as mean ± SD of three independent experiments (*n* = 3) and normalized to the GADPH signal. Different superscript letters between different strawberry dried methanolic extract concentrations for each assayed protein indicate statistical significance (*p* < 0.05). p-AMPK: phosphorylated AMP-activated protein kinase; AMPK: AMP-activated protein kinase; Sirt1: sirtuin 1; LKB1: liver kinase B1; GADPH: glyceraldehyde 3-phosphate dehydrogenase.

To investigate whether strawberry extract regulates metabolism in HepG2 cells via AMPK pathway, the protein levels of phosphorylated AMPK (p-AMPK), total AMPK, sirtuin1 (Sirt1), and liver kinase B1 (LKB1) were examined. As demonstrated in Figure 2, treatment with strawberry dried methanolic extract significantly (*p* < 0.05) increased the expression of all these proteins in a dose dependent manner.

Twenty-four hours of treatment with the highest concentration (100 µg/mL) of strawberry dried methanolic extract, led to a 3.06-, 2.37-, and 3.16-fold overexpression of p-AMPK/AMPK, Sirt1, and LKB1, respectively, compared to control.

**Figure 3:**
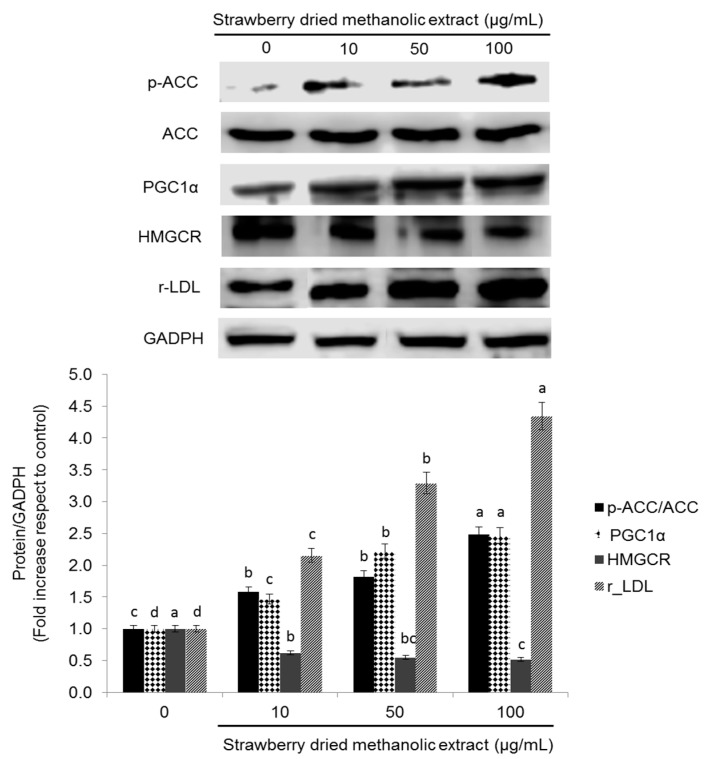
Effects of strawberry dried methanolic extract on p-ACC, ACC, r-LDL, PGC-1α, and HMGCR expression. HepG2 cells were treated with the indicated concentrations for 24 h. The protein signals were detected by a Lycor C-Digit Blot Scanner and quantification was made using the software Image Studio 3. Values are expressed as mean ± SD of three independent experiments (*n* = 3) and normalized to the GADPH signal. Different superscripts letters between different strawberry dried methanolic extract concentrations for each assayed protein indicate statistical significance (*p* < 0.05). p-ACC: phosphorylated acetyl coenzyme A carboxylase; ACC: acetyl coenzyme A carboxylase; PGC-1α: peroxisome proliferator activated receptor gamma coactivator 1-alpha; HMGCR: 3-hydroxy-3-methylglutaryl-CoA reductase; r-LDL: low density lipoprotein receptor; GADPH: glyceraldehyde 3-phosphate dehydrogenase.

Activation of AMPK by strawberry dried methanolic extract was accompanied by an overexpression of the inactive form of the ACC (phosphorylated ACC) and upregulation of the peroxisome proliferator activated receptor gamma coactivator 1-alpha (PGC-1α)—another downstream target of AMPK—in a dose dependent manner (Figure 3). p-ACC/ACC expression was 1.58-, 1.82-, and 2.48-fold, and PGC-1α expression 1.47-, 2.22-, and 2.47-fold higher compared to the control when cells were treated with 10, 50, and 100 µg/mL of the strawberry dried methanolic extract, respectively.

In addition, the expression of HMG-CoA reductase (HMGCR), which catalyzes a rate limiting step in cholesterol synthesis, was suppressed by the strawberry treatment (Figure 3). HMGCR expression was significantly (*p* < 0.05) lower (from 0.62 to 0.52 times compared to the control) when strawberry extract was applied at 10 µg/mL or 50–100 µg/mL, respectively. Inhibition of HMGCR and ACC confirms the hypothesis that strawberry extracts should have positive effects in lipid metabolism in HepG2 cells.

In order to address whether strawberry extracts can influence lipid metabolism through transcriptional regulation, the expression of LDL receptor (r-LDL) was investigated. Dried methanolic extract at 10, 50, and 100 µg/mL led to significantly (*p* < 0.05) higher r-LDL expression compared to the control by up to 2.15-, 3.29-, and 4.34-fold, respectively.

**Figure 4:**
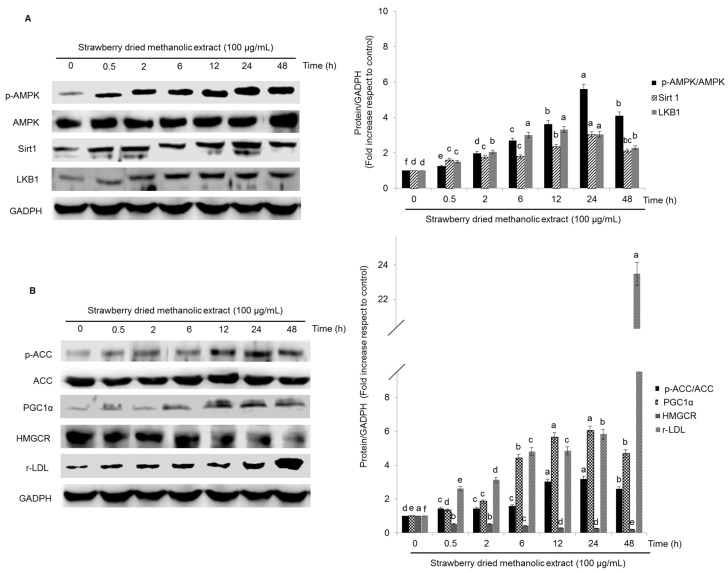
Protein ((**A**): p-AMPK, AMPK, Sirt1, LKB1 and (**B**): p-ACC, ACC, PGC-1α, HMGCR, r-LDL) expression in HepG2 cells after treatment with strawberry dried methanolic extract for different times. Cells were treated with 100 µg/mL of the extract. The protein signals were detected by a Lycor C-Digit Blot Scanner. p-AMPK: phosphorylated AMP-activated protein kinase; AMPK: AMP-activated protein kinase; Sirt1: sirtuin 1, LKB1: liver kinase B1; p-ACC: phosphorylated acetyl coenzyme A carboxylase; ACC: acetyl coenzyme A carboxylase; PGC-1α: peroxisome proliferator activated receptor gamma coactivator 1-alpha; HMGCR: 3-hydroxy-3-methylglutaryl-CoA reductase; r-LDL: low density lipoprotein receptor; GADPH: glyceraldehyde 3-phosphate dehydrogenase.

The effects of strawberry extract in the expression of all the proteins tested was also time-dependent (Figure 4A,B). In most cases (p-AMPK, Sirt1, LKB1, p-ACC, PGC-1α), the highest protein expression was observed after 24 h of treatment, although, the first significant effects (*p* < 0.05) were observed just after 30 min. Only in the case of r-LDL and HMCGR, the maximal activation/inhibition was obtained after 48 h, respectively.

**Figure 5:**
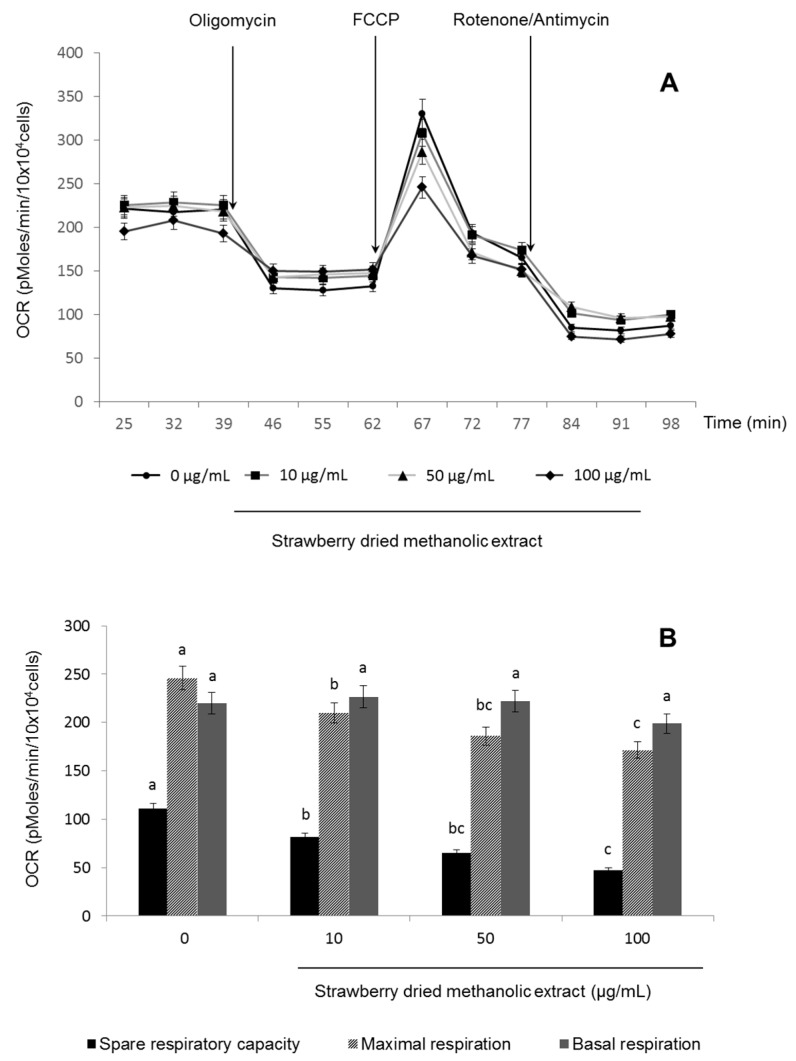
Effects of strawberry dried methanolic extract on mitochondrial respiration in HepG2 cells: XF cell Mito stress test profile (**A**) and spare respiratory capacity, maximal respiration and basal respiration (**B**). Cells were treated with the indicated concentration for 24 h. Values are expressed as mean ± SD of three independent experiments (*n* = 3). Different superscript letters between different strawberry dried methanolic extract concentrations for each parameter in the chart bar indicate statistical significance (*p* < 0.05). OCR: oxygen consumption rate; FCCP: carbonyl cyanide-4-(trifluoromethoxy)phenylhydrazone.

Since AMPK is activated not only through its upstream kinases but also by an increase in the AMP/ATP ratio or in response to other stimuli such as hypoxia or oxidative stress [9,35,36], mitochondrial functionality in treated HepG2 cells was also determined. For that purpose, the OCR was examined (Figure 5A).

Increasing concentrations of strawberry dried methanolic extract had no significant (*p* < 0.05) effect on basal respiration, while they caused a diminution in the spare and the maximal respiration compared to the control (Figure 5B).

To further confirm the effect of strawberry extracts on lipid metabolism through AMPK pathway activation, HepG2 cells were incubated with 100 µg/mL strawberry dried methanolic extract in the presence of an AMPK inhibitor (compound C, 10 mM) or a positive control (lovastatin, 10 mM). Then, the expression of AMPK pathway related proteins was determined.

**Figure 6:**
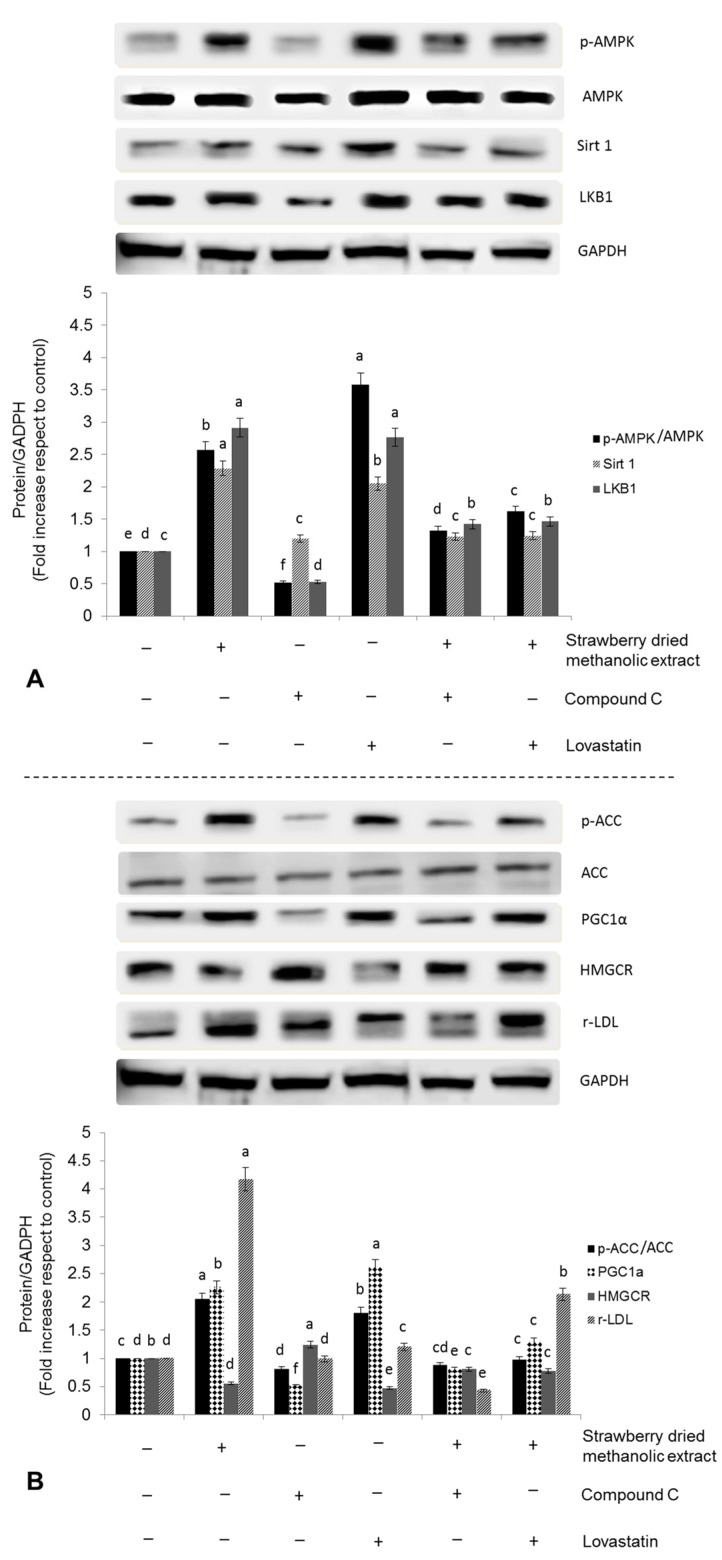
Effects of strawberry dried methanolic extract (100 µg/mL) in the expression of AMPK pathway related proteins ((**A**): p-AMPK, AMPK, Sirt1, LKB1 and (**B**): p-ACC, ACC, PGC1α, HMGCR, r-LDL) in the presence of 10 µM Compound C (negative control) or 10 µM Lovastatin (positive control) in HepG2 cells. Cells were treated for 24 h. Values are expressed as mean ± SD of three independent experiments (*n* = 3). Different superscript letters between different assayed conditions for each assayed protein indicate statistical significance (*p* < 0.05). p-AMPK: phosphorylated AMP-activated protein kinase: AMPK: AMP-activated protein kinase; Sirt1: sirtuin 1; LKB1: liver kinase B1; p-ACC: phosphorylated acetyl coenzyme A carboxylase; ACC: acetyl coenzyme A carboxylase; PGC-1α: peroxisome proliferator activated receptor gamma coactivator 1-alpha; HMGCR: 3-hydroxy-3-methylglutaryl-CoA reductase; r-LDL: low density lipoprotein receptor; GADPH: glyceraldehyde 3-phosphate dehydrogenase.

As shown in Figure 6A,B, phosphorylation of AMPK by strawberry dried methanolic extract was significantly (*p* < 0.05) reversed in the presence of compound C, reaching values comparable to the untreated control.

Also, the expression of p-ACC, r-LDL, and PGC-1α was lower in the presence of the inhibitor, supporting the role of AMPK in the activation of these proteins by strawberry extracts, at least in part. Inhibition of HMGCR induced by strawberry extracts was also significantly (*p* < 0.05) reversed by compound C treatment.

On the contrary, and as expected, treatment with 10 µM lovastatin for 24 h led to a higher expression of p-AMPK, with the corresponding implications for its downstream targets. It has been demonstrated that lovastatin treatment (1–25 µM, 24 h) impairs mitochondrial function, decreases cellular ADP/ATP ratios, and consequently activates LKB1/AMPK pathway [37]. Statins can rapidly activate AMPK via increased Thr-172 phosphorylation in vitro and in vivo [38,39,40].

It was interesting that no synergistic effects were observed when the cells were simultaneously treated with lovastatin and strawberry dried methanolic extract.

### 3.4. Effects of Strawberry Extract on Lipid Profile

**Figure 7:**
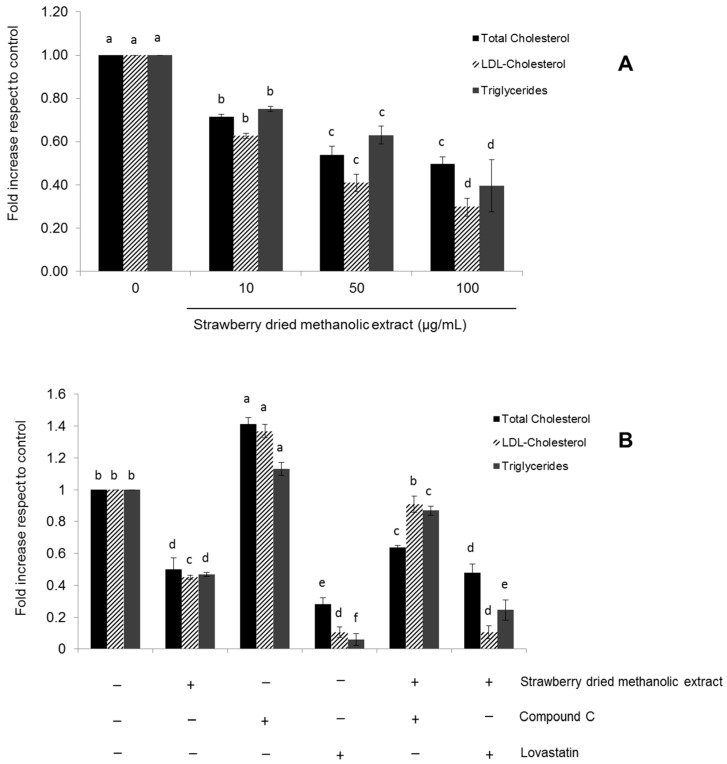
Effects of strawberry dried methanolic extract on lipid profile (total cholesterol and LDL-cholesterol and triglycerides) in HepG2 cells. (**A**) Cells were treated with the indicated concentration of the extract for 24 h. (**B**) Cells were treated with 100 µg/mL of the extract in the presence of 10 µM Compound C (negative control) or 10 µM Lovastatin (positive control) for 24 h. Values are expressed as mean ± SD (*n* = 3) of three independent experiments. Different superscript letters between different assayed conditions for each marker (total cholesterol, LDL-cholesterol, or triglycerides) indicate statistical significance (*p* < 0.05).

The effects of the strawberry extract in lipid metabolism in HepG2 cells were confirmed by determining total cholesterol, LDL-cholesterol, and triglycerides content after treatment for 24 h. As shown in Figure 7A, strawberry dried methanolic extract significantly (*p* < 0.05) improved the lipid profile in a dose dependent manner.

Total cholesterol, LDL-cholesterol, and triglycerides levels were lower (up to 0.50-, 0.30-, and 0.40-fold) compared to control when cells were treated with the higher concentration of the extract (100 µg/mL).

The same determinations were performed in the presence of compound C or lovastatin (Figure 7B). In cells treated simultaneously with compound C and strawberry dried methanolic extract, the total cholesterol, LDL-cholesterol, and triglycerides levels were, respectively, 1.27, 2.01, and 1.86 times higher compared to cells treated exclusively with the strawberry extract (100 µg/mL). The highest lipids content was observed in cells treated exclusively with the AMPK inhibitor, while the best results were observed after treatment with lovastatin. 

**Figure 8:**
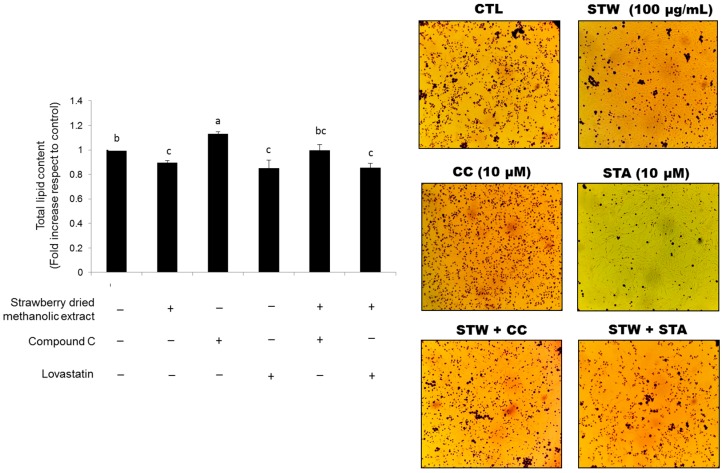
Effects of strawberry dried methanolic extract (100 µg/mL) on total lipid accumulation in HepG2 cells in the presence of 10 µM Compound C (negative control) or 10 µM Lovastatin (positive control). Cells were treated for 24 h. Stained intercellular oil droplets were eluted with isopropanol and quantified by spectrophotometrical analysis at 510 nm. Values are expressed as mean ± SD of three independent experiments (*n* = 3). Columns with different superscript letters are significantly different (*p* < 0.05). CTL: untreated cells; STW: cells treated with strawberry dried methanolic extract; CC: compound C; STA: lovastatin.

Also, the total lipid accumulation determined by O red oil staining was significantly higher (*p* < 0.05) in cells treated with the inhibitor of AMPK activation and almost similar in control cells compared to the cells treated simultaneously with compound C and strawberry extract (Figure 8).

## 4. Discussion

Beneficial effects of strawberry consumption comprise a wide range of biological activities, from antioxidant capacity to anti-inflammatory, anti-hypertensive, and anti-proliferative actions [11,12,19,41,42,43]. Epidemiological and clinical studies indicate that acute consumption of strawberry improves postprandial glycemic response, circulating inflammatory markers, and increases plasma antioxidant capacity. Likewise, a sustained strawberry consumption ameliorates plasma lipid profile and decreases chronic inflammation, mainly in subjects with high risk for metabolic syndrome. These beneficial effects have been frequently attributed to the antioxidant capacity of strawberry bioactive compounds. However, it has been recently suggested that strawberry’s biological properties are also related to the modulation of several molecular pathways implicated in cell proliferation, survival, and metabolism [43]. In this report, we showed for the first time that the effects of strawberry extract in lipid metabolism are related to the AMPK pathway.

AMPK complex has a direct impact on glucose and lipid metabolism improving glucose uptake and inhibiting fat accumulation through the modulation of several downstream-signaling components like the ACC [9,10]. Thus, pharmacological activation of AMPK in the liver would be expected to improve lipid profile and to decrease the incidence of dyslipidemia related diseases.

Our data illustrate that strawberry dried methanolic extract led not only to higher AMPK phosphorylation but also to a higher expression of LKB1 and Sirt1 as well. LKB1 was the first upstream kinase of AMPK phosphorylation to be described, while Sirt1 is a histone/protein deacetylase that cross-regulates AMPK [36]. Evidence indicates that both Sirt1 and AMPK mutually affect lipid metabolism, but their relationship in the signaling cascade appears to be uncertain [44,45].

The AMPK activation through LKB1 stimulation in HepG2 cells has been previously reported for other natural compounds but not for strawberries. For example, Kim et al. [46] reported that thymoquinone relieves the inflammatory response and hepatic fibrosis through the activation of the LKB1/AMPK signaling pathway, and honokiol, a bioactive compound obtained from the stem bark of *Magnolia officinalis*, protects against liver injury caused by hypoxia/reoxygenation and hepatotoxicants via an LKB1-dependent pathway [47].

Induction of Sirt1 is also related to the expression of PGC-1α that turned out to be stimulated by strawberry extract treatment in a dose-dependent manner. Sirt1 can directly interact and deacetylate PGC-1α, which is closely related with enhanced PGC-1α transcriptional activation. Sirt1-mediated regulation of PGC-1α activity may play a major role in the metabolic adaptations to energy metabolism in different tissues [48]. In liver, Sirt1 regulates gluconeogenic activity by modulating cAMP responsive element binding protein regulated transcription coactivator 2 and PGC-1α, which is also an important mediator of AMPK-induced gene expression. In that sense, AMPK activation increases PGC-1α expression at the same time as it requires PGC-1α activity to regulate the expression of several key players in glucose and mitochondrial metabolism [49,50,51]. Therefore, the relation between AMPK and PGC-1α connects the sensing of the cellular energetic status and the stimulation of transcriptional programs that control energy expenditure [51].

Furthermore, in the present study, strawberry extract led to a higher phosphorylation of the AMPK substrate ACC. Phosphorylation of ACC leads to its inactivation and consequently to the acute interruption of fatty acid synthesis by blocking the conversion of acetyl CoA to malonyl CoA. In addition, the ACC inhibition could also increase β-oxidation by promoting the activity of carnitine palmitoyltransferase (CPT1), which is essential for the entry of long-chain fatty acids into mitochondria.

Strawberry extract also inhibited HMGCR and stimulated r-LDL, which is subjected to sensitive feedback control that regulates intracellular and extracellular cholesterol levels. When cholesterol levels rise within the cell, r-LDL production decrease contemporarily with the HMGCR diminution, leading to a reduction of cholesterol input from both plasma and endogenous synthesis. On the contrary, in cholesterol depleted cells the production of r-LDL and HMGCR are increased. The mechanism for this dual regulation is controlled by the sterol regulatory element-binding protein (SREBP). In cholesterol depleted cells, SREBP activates HMGCR, r-LDL, and all the other enzymes implicated in cholesterol biosynthesis, at a transcriptional level. When LDL-derived cholesterol enters the cells, it blocks the proteolytic release of the active fragment of SREBP from membranes, leading to a failure in the transcription of the target genes, which in turn decreases cells cholesterol biosynthesis and prevents cholesterol overload [52].

SREBP-mediated regulation of LDL receptors is essential for the action of statins, a class of plasma cholesterol lowering drugs which have been found to reduce CVDs and mortality in individuals at high risk. In the liver, statins are capable of inhibiting HMGCR and subsequently reducing the cholesterol production. This cholesterol diminution activates SREBP-mediated regulation resulting in an increased number of r-LDL on cell membranes and an increased amount of HMGCR. Nevertheless, cholesterol biosynthesis is not stimulated due to the inhibitory effect of statin on the enzyme. The newly produced r-LDL translocate LDL from the blood into the cell, where its transformation releases cholesterol that becomes available for metabolic purposes. The overall effect is that the amount of cholesterol in the liver remains at a normal level while the level of LDL-cholesterol in the blood is maintained low [52]. Similar effects to those described for statins could explain the effects reported for strawberries in the present work concerning cell cholesterol levels. In fact, our results showed that both strawberry extract and lovastatin presented similar effects on lipid profile, cell lipid accumulation, and on the expression of the proteins related to the AMPK pathway. Interestingly, no synergistic effects on the expression levels of such proteins were observed when cells were treated with strawberry extract and lovastatin simultaneously. We hypothesized that strawberry extract may interfere with the cellular uptake of the drug and/or its metabolism. For example, it is well known that some Cytochrome P450 3A4 inducers may reduce the efficacy of lovastatin. On the contrary, the positive effects of the strawberry extract were remarkably inhibited in the presence of the AMPK-pharmacological inhibitor compound C, demonstrating that activation of the AMPK pathway is needed, at least in part, for the strawberry effects on cell lipid metabolism.

Finally, strawberry extract also caused a diminution on the spare and the maximal respiratory capacity. The spare, or reserve respiratory capacity, describes the amount of extra ATP that can be produced by oxidative phosphorylation in case of a rapid increase in energy demand. The energy requirement of different tissues fluctuates constantly and the ATP metabolism is correspondingly regulated to avoid futile energy expenditure and to provide their specific needs. Concomitantly, the mitochondrial respiration rate augments when more ATP is synthetized by mitochondria, underlying the link between cellular ATP demand and oxidative phosphorylation regulation [53]. Therefore, a decrease in the spare respiratory capacity and consequently a disturbance in the cellular energy state, as described after the strawberry extract treatment in the present study, could lead to AMPK activation. However further analyses should be performed to confirm that the AMP/ATP ratio certainly increases.

Usually, reduced mitochondrial function has been linked to the pathogenesis of numerous diseases and their complications. However, some authors [53] have recently suggest that overstimulation of mitochondria is a probable risk factor for insulin resistance, while moderate mitochondrial dysfunction may actually be protective under certain conditions, suggesting the mitochondrial modulation as a prospective therapy for metabolic diseases. In fact, it has been demonstrated that several mitochondrial modulators ameliorate insulin sensitivity and metabolic complications [54,55,56]. For example, it has been reported that metformin—which is widely used for the treatment of type 2 diabetes—directly inhibits complex I of the electron transport chain (ETC) and thus reduces mitochondrial respiration [53,57,58,59]. Therefore, it can be hypothesized that strawberry extract activates AMPK not only via LKB1 but also through alterations on the electron transport chain functionality, subsequently affecting cell energy state. Similar results have been reported for berberine [60] and licochalcone A [61].

Activation of AMPK by strawberry methanolic extract could also be related to its antioxidant capacity, since the AMPK pathway is implicated in the endogenous antioxidant response of the organism through the activation of the nuclear related factor 2 and consequently of some antioxidant responsive elements [62,63,64].

## 5. Conclusions

In summary, our results provide new insights into the molecular mechanism of the hypocholesterolemic effects of strawberry extract. Strawberry treatment activated the energy sensing molecule AMPK, which in turn inhibited the proteins involved in fatty acids and cholesterol synthesis. These findings support the further employment of strawberry fruits as functional foods and a potential hypolipidemic agent.

## Figures and Tables

**Table 1:** Phytochemical composition and antioxidant capacity of strawberry fruit and methanolic extract.

Parameters	Fresh Fruit	Dried Methanolic Extract
Total Polyphenols (mg GAeq/g)	2.64 ± 2.63	23.44 ± 0.22
Total Flavonoids (mg CATeq/g)	1.02 ± 0.87	5.21 ± 0.29
Vitamin C (mg/g)	0.39 ± 0.23	9.09 ± 4.45
Cyanidin-3-*O*-glucoside (mg/g)	0.03 ± 0.02	0.69 ± 0.41
Pelargonidin-3-*O*-glucoside (mg/g)	0.70 ± 0.25	16.32 ± 5.71
Pelargonidin-3-*O*-rutinoside (mg/g)	0.04 ± 0.08	0.93 ± 1.39
**TAC (µmol Txeq/g)**		
FRAP	22.70 ± 2.03	168.25 ± 3.95
DPPH	8.11 ± 0.25	30.29 ± 0.18
TEAC	10.71 ± 0.58	35.51 ± 0.06

mg GAeq/g: mg of gallic acid equivalent/g of fresh fruit or dried strawberry methanolic extract; mg CATeq/g: mg of catechin equivalent/g of fresh fruit or dried strawberry methanolic extract; µmol Txeq/g: µmol of Trolox equivalent/g of fresh fruit or dried strawberry methanolic extract; TAC: total antioxidant capacity; FRAP: Ferric Reducing Antioxidant Power; DPPH: 2,2-diphenyl-1-picrylhydrazyl; TEAC: Trolox Equivalent Antioxidant Capacity.
